# Analytical computation for segmentation and classification of lumbar vertebral fractures

**DOI:** 10.3389/fncom.2025.1536441

**Published:** 2025-07-10

**Authors:** Roseline Nyange, Hemachandran Kannan, Channabasava Chola, Saurabh Singh, Jaejeung Kim, Anil Audumbar Pise

**Affiliations:** ^1^Department of Computer Engineering, Jeonbuk National University, Jeonju, Republic of Korea; ^2^Department of Business Analytics, School of Business, Woxsen University, Hyderabad, India; ^3^Department of Studies in Computer Science, University of Mysore, Mysuru, Karnataka, India; ^4^Department of AI and Big Data, Woosong University, Daejeon, Seoul, Republic of Korea; ^5^Department of Computer Science and Engineering, Chungnam National University, Daejeon, Republic of Korea; ^6^X-idian, Johannesburg, South Africa

**Keywords:** classification, MRI, segmentation, vertebral body compression fractures, feature based classification

## Abstract

Spinal health forms the cornerstone of the overall human body functionality with the lumbar spine playing a critical role and prone to various types of injuries due to inflammation and diseases, including lumbar vertebral fractures. This paper proposes automated method for segmentation of lumbar vertebral body (VB) using image processing techniques such as shape features and morphological operations. This entails an initial phase of image preprocessing, followed by detection and localizing of vertebral regions. Subsequently, vertebral are segmented and labeled, with each classified into normal or fractured using classification techniques, k-nearest neighbors (KNN) and support vector machines (SVM). The methodology leverages unique vertebral characteristics like gray scales, shape features, and textural elements through a range of machine learning methods. The approach is assessed and validated on a clinical spine dataset dice score used for segmentation, achieving an average accuracy rate of 95%, and for classification, achieving average accuracy of 97.01%.

## 1 Introduction

Medical imaging offers an opportunity to observe the internal structure of human anatomy .Integrating artificial intelligence (AI) techniques into healthcare has driven significant innovations, offering considerable promise in advancing medical practices and improving diagnostic accuracy (Galić et al., 2023). The merging of AI with medical image analysis marks a pivotal achievement, offering deep insights into human anatomy and physiology by enabling advanced visual data interpretation (Mohammed et al., 2022). The combination of computational intelligence with medical imaging has driven the evolution of advanced techniques crucial for disease detection, prognosis, and treatment planning. It can be effectively used in clinical settings to identify and choose appropriate treatments for abnormalities, in research to gain insights and develop novel medications for various conditions, and in surgical planning for guidance.

Vertebral fractures occur when the vertebral body, which is front part of the spinal bone, cannot support the load of the spine from above.Vertebral fractures affect many patients worldwide and are most common in elderly population, these affects their daily life due to severe physical limitations (Akeda et al., 2024). In clinical routine, it is identified as partial collapse of affected vertebral body. Vertebral fracture causes changes in shape of vertebrae which may also occur with deformation of vertebral plateaus. When a patient develops a vertebral collapse without apparent trauma it needs to be investigated (Parizel et al., 2010). Vertebral fractures (VF) most frequently occur in the vertebral body, often resulting from osteoporosis in adults or trauma in children, as well as from infections or tumors, all of which can lead to compression fractures (Frighetto-Pereira et al., 2015) . These fractures are prevalent in the thoracolumbar spine (middle-lower spine) and can cause a reduction in height if multiple fractures occur. In adults, these fractures typically indicate osteoporosis, whereas, in children, they are usually caused by trauma. Spinal fractures elevate the risk of future spinal fractures and other low-impact fractures elsewhere in the body (Eastell et al., 2001). Although all fractures cause deformities, not all deformities arise from fractures. Clinically, a vertebral compression fracture due to osteoporosis is often considered benign, while one caused by bone metastasis is typically regarded as malignant (Cicala et al., 2013).

Medical institutions and hospitals generate and process a large volume of medical images every day. The manual analysis of these images demands considerable expertise and is time-intensive and expensive. Consequently, the demand for automated medical image processing in the medical field is increasing. MRI offer significant advantage over other imaging techniques like CT and X-ray due to absence of radiation exposure during scans (Lee et al., 2012) and the ability to effectively address certain pathologies such as bone tumors and metastases. These advantages makes MRI a frequently utilized imaging modality in diagnosis of spinal diseases and abnormalities such as vertebral fractures, slipped vertebrae, herniated or degenerated discs, vertebral deformities and bone marrow deformities (Bot et al., 2004).

Computer-Aided Diagnosis (CAD) systems have become essential tools for evaluating anatomical structures in medical images, facilitating the diagnosis of pathologies, vertebral fractures, tumors, and abnormalities. They also support treatment planning, surgical interventions, and post-surgical assessments (Song et al., 2016). Despite their advantages, the application of CAD systems to spinal MRI faces significant challenges. MRI scans inherently differ from modalities like computed tomography (CT) because they do not use standardized quantitative units equivalent to Hounsfield Units (HU), which measure tissue density in CT scans (Pinto et al., 2022). This absence of standardized measurement units makes MRI interpretation more subjective and challenging. Furthermore, spinal MRIs are complicated by issues such as arbitrary fields of view, variable image resolutions, and susceptibility to image artifacts (Chang et al., 2020). Accurate vertebral localization, labeling, and segmentation in MRI is particularly challenging due to the complex vertebral anatomy and anatomical variability among patients, exacerbated by conditions such as scoliosis and vertebral compression fractures (Alomari et al., 2015). Vertebral fractures, a common indicator of osteoporosis and metastatic diseases, demands precise imaging for diagnosis and treatment planning. While conventional radiography (X-ray), including advanced low dose EOS imaging, is frequently employed as an initial evaluation method due to its accessibility, rapid imaging, and reduced radiation exposure however, its sensitivity in identifying subtle or early-stage vertebral abnormalities remains limited (Garg et al., 2020) . Conversely, MRI remains the preferred modality for detailed evaluation of vertebral fractures because of its superior capability to visualize marrow changes, edema, soft tissue involvement, and differentiate benign from malignant vertebral fractures, highlighting the critical need for an effective CAD system.

This paper is organized into six sections. II. Literature Review reviews the relevant literature, summarizing previous research and foundational studies. III. Methodology details the materials and techniques used in this study. IV. Experimental Setup outlines the experimental setup and presents the results, providing a thorough evaluation of the findings. V. Discussion discusses the results, interpreting the data and considering its implications. Finally, VI. Conclusion concludes the paper, summarizing the main findings and suggesting directions for future research and potential applications.

## 2 Literature review

Vertebral compression fractures (VCFs) may arise either from traumatic events, such as falls or vehicular accidents, or due to underlying pathological conditions compromising bone integrity. Osteoporosis, characterized by a significant decline in bone density, makes vertebrae particularly susceptible to fractures. Resultant VCFs often lead to chronic pain, spinal deformities, and noticeable height loss, severely impairing daily functioning and quality of life, and increasing dependence on assisted care (Haffner et al., 2021; Pisani et al., 2016). The diagnosis process of VCFs involves segmentation and classification. Segmentation methods can be categorized as manual, semi-automatic, or automatic. Manual segmentation, while accurate, is labor-intensive and prone to operator variability. Azevedo-Marques et al. (2015) proposed a manual segmentation using Adobe Photoshop CS5^TM^ software for precise delineation of vertebral boundaries but required significant operator input. To mitigate manual method limitations, semi-automatic segmentation techniques have emerged. Kim et al. (2018) suggested semi-automatic algorithm for the segmentation of vertebral bodies in magnetic resonance (MR) the placement of Region of Interest (ROI) limited to a single area containing a vertebral body. The correlation algorithm subsequently identified the other vertebral bodies, facilitating the segmentation process via graph-based and line-based segmentation algorithms.When tested on sagittal MR images of the lumbar spine, this method achieved a Dice Similarity Coefficient (DSC) of 90%.

Automatic segmentation techniques have seen rapid advancements using deep learning approaches. Lessmann et al. (2019) proposed a vertebra segmentation and identification method using a single fully convolutional neural network (FCN) for multiple tasks. The method employs a patch-based approach, where each patch contains at least one vertebra. After segmenting a vertebra, anatomical knowledge about typical vertebral positions is used to reposition the patch for segmenting the next vertebra, ensuring spatial continuity. This approach resulted in an average (DSC) of 94.9 ± 2.1%, and an anatomical identification accuracy of 93%, corresponding to just one mislabeled vertebra. Vertebrae visibility classification reached an accuracy of 97%. Song et al. (2024) highlight the importance of effectively utilizing AI-generated annotations, on combating annotation errors in existing methods for medical image segmentation, opening the opportunity to use AI-generated annotations to train segmentation model for medical image segmentation.

Following segmentation, classification techniques distinguish between normal, benign or malignant vertebral fractures based on extracted image features. Azevedo-Marques et al. (2015) proposed classification of malignant vs. benign VCFs in magnetic resonance imaging (MRI) using contrast and texture features and a k-nearest neighbor (KNN) classifier results demonstrated that by combining features derived from Fourier and wavelet transforms with the fractal dimension, a classification accuracy of 94.7% was achieved. Frighetto-Pereira et al. (2016) extended this by integrating texture and shape features, achieving an area under the receiver operating characteristic curve (AUC) of 0.97 for differentiating normal from fractured vertebral bodies, and 0.92 between benign and malignant fractures. These results were achieved using the k-nearest neighbor method, a neural network with radial basis functions, and a naïve Bayes classifier, all applied with feature selection. Similarly, Arpitha and Rangarajan (2021) proposed an approach utilizing texture and shape features with data augmentation techniques, reporting classification accuracies ranging from 92.3% to 96.07% for various fracture categories.

Further advancements leverage deep learning and neural networks. Tomita et al. (2018) combining deep residual networks (ResNet) and recurrent neural networks (RNN) is used for feature extraction and aggregation. ResNet processed and extracted features from two-dimensional (2D) CT slices. These features were then input into a long short-term memory (LSTM) network, part of the RNN module, which aggregated information from multiple slices to make the final diagnosis.This method achieved an accuracy of 89.2% and an F1 score of 90.8% in evaluation, conducted on a held-out test set comprising 129 CT scans. Germann et al. (2023) proposed a deep convolutional neural network (DCNN) for automated vertebral measurements and insufficiency fracture detection on lumbar spine MRI. Using a U-Net-based architecture, the model achieved excellent agreement with radiologists (ICC > 0.94) and high diagnostic accuracy (94% sensitivity, 97% specificity). Its performance was consistent across scanners and field strengths, highlighting its potential for clinical application. Yeh et al. (2022) developed a deep learning decision support model based on the ResNet50 architecture to differentiate benign from malignant spinal fractures on MRI. Using T1- and T2-weighted slices from 190 patients, the model achieved 92% accuracy and significantly improved the diagnostic performance of a first-year resident sensitivity from 78% to 94%, specificity from 61% to 91%. The study demonstrates the potential in of support less experienced clinicians. Another line of work explores fuzzy-image-fusion classifiers to handle uncertainty and noise in medical imaging. Versaci et al. (2025) organize similar eddy-current defect maps into coherent, class-specific sets by treating similarity as the complement of a fuzzy distance measure in feature space. Because MRI segmentation often suffers from noise and subtle pathological variations, adopting fuzzy-similarity-based classifiers–such as the image-fusion approach yield robustness to artifacts and improve classification.

The diagnosis of vertebral compression fractures (VCFs) stems from the critical need for precise vertebral body (VB) positional angles and alignment of adjacent vertebrae, surface appearance analysis and spatial relationships between vertebrae and other spinal structures to improve segmentation and fracture classification. These elements are essential for enhancing the diagnostic accuracy of clinicians. This study is motivated by the need to develop a robust clinical decision support system that reliably segments and classifies VCFs while addressing these challenges. Our approach strategically integrates machine learning (ML) techniques such as K-Nearest Neighbors (KNN) and Support Vector Machines (SVM) with carefully selected features, thereby capturing critical anatomical details. Deep learning methods, while powerful, require extensive, well-annotated datasets and significant computational resources. Given our dataset size, ML methods reduce the risk of overfitting and ensuring computational efficiency.

The contribution of our work is as follows, first we introduce a resource-efficient segmentation pipeline that combines spatial filtering, adaptive histogram equalization, median-filter smoothing, morphological operations and convex-hull regularization to facilitate precise segmentation of lumbar vertebral fracture in MRI. Second, we design a compact yet discriminative feature set drawing on shape features (eccentricity, rectangularity, convexity), grayscale and texture descriptors, and we integrate synthetic oversampling (SMOTE) to address class imbalance in clinical data. Third, we conduct comparison of K-Nearest Neighbors and Support Vector Machines demonstrating that our SVM-based classifier achieves high accuracy. Together, these contributions yield an automated VCF decision-support system that balances diagnostic performance with computational efficient and deployable in resource-limited clinical settings.

## 3 Methodology

**Figure 1:**
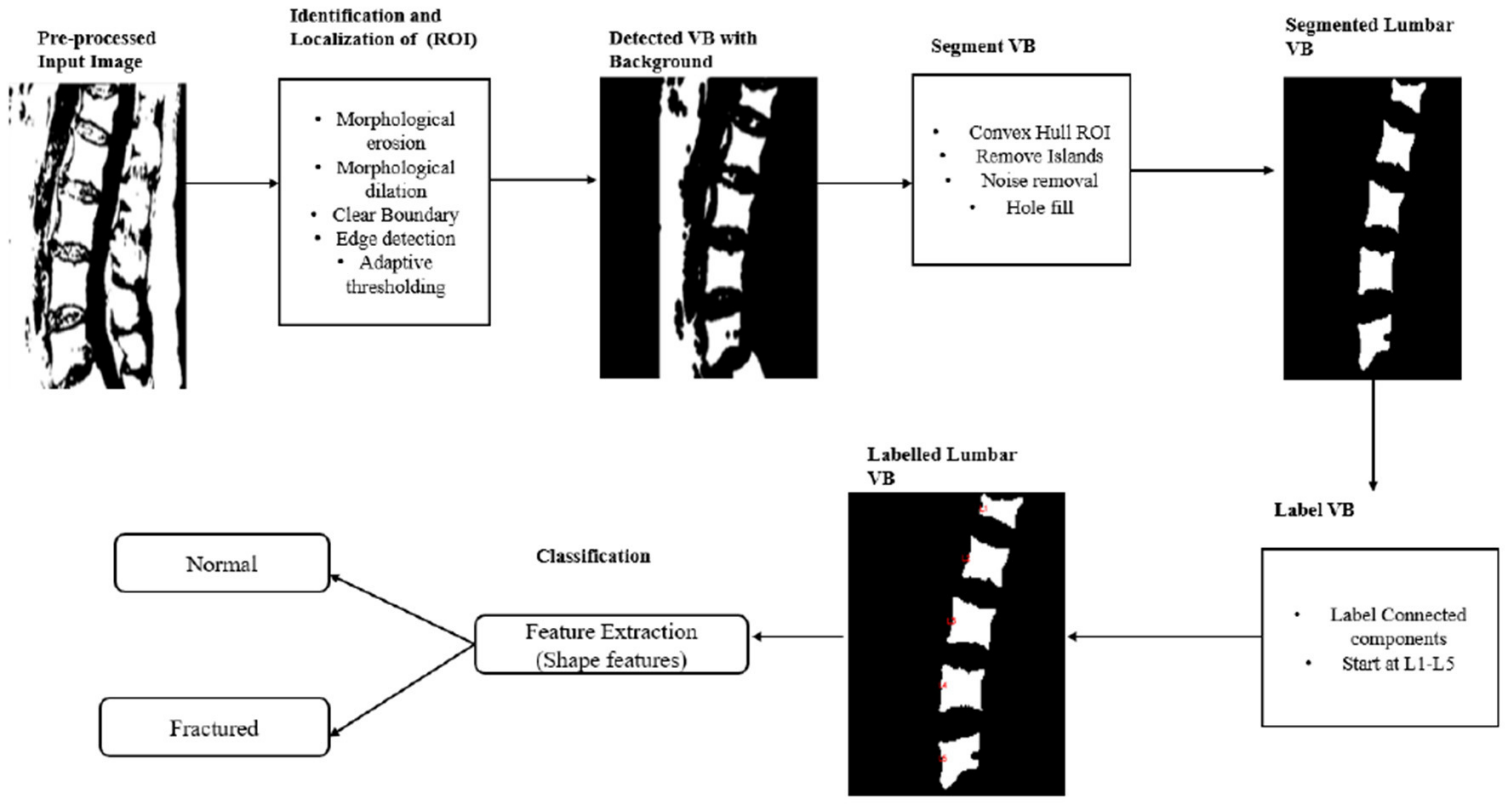
Methodology workflow.

This study presents a framework for segmentation and classification aimed at distinguishing bone from soft tissue in MR images. The process, which spans from an initial MRI T1-weighted median sagittal slices scan to the radiological assessment for vertebral fracture classification, comprises three sequential steps, the detailed steps are as follows: (i)data preprocessing; (ii) segmentation of the lumbar vertebral bodies (VBs); and (iii) feature extraction from lumbar VBs to classify each VB as either normal or fractured. Figure 1 illustrates the workflow of this method.

### 3.1 Data and preprocessing

**Figure 2:**
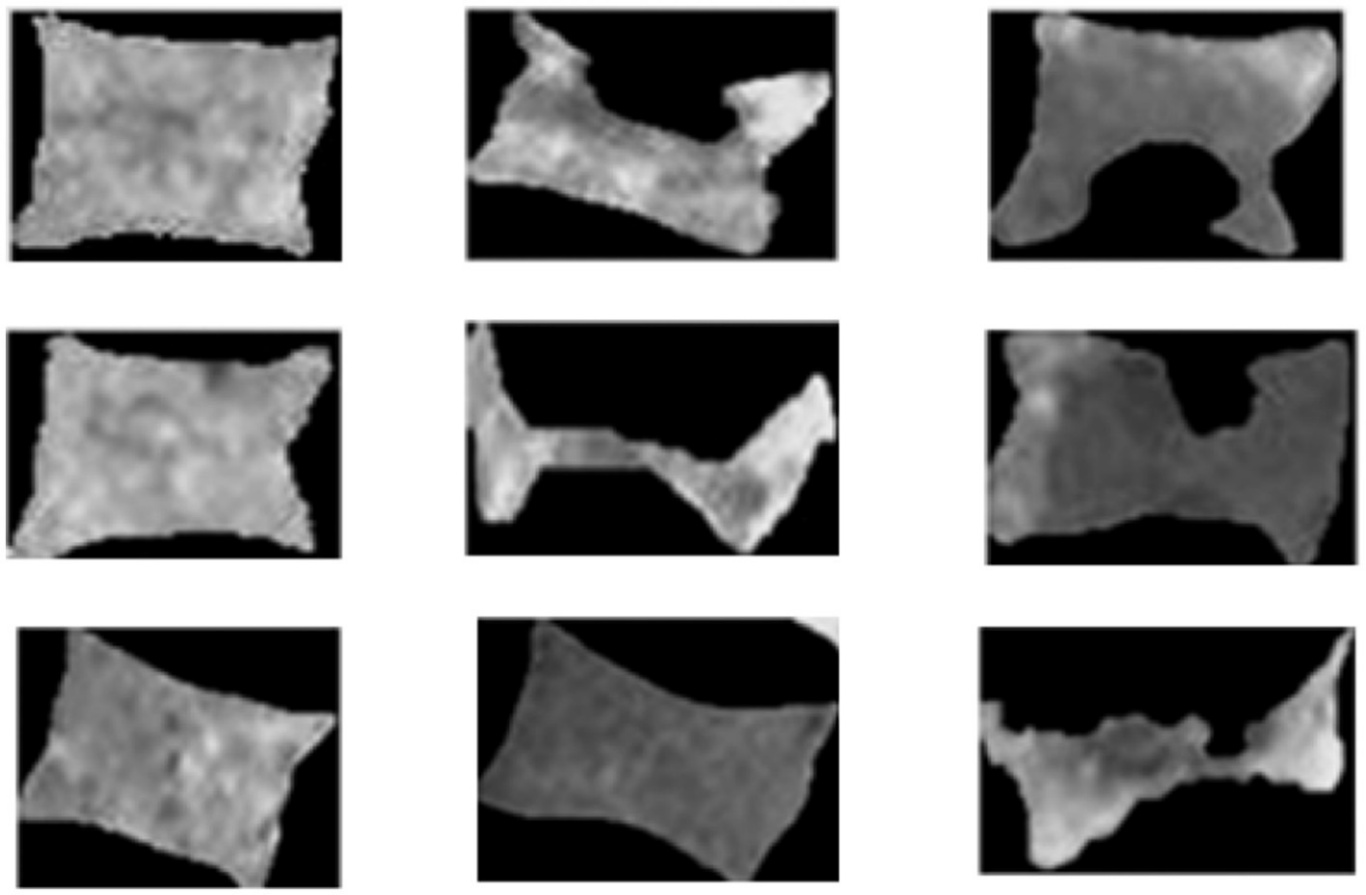
Instances of lumbar vertebral bodies (VBs) showing normal and fractured conditions. Source: Frighetto-Pereira et al. (2016).

The dataset, sourced from Frighetto-Pereira et al. (2016), includes 63 patients (37 women, 26 men) with a mean age of 62.25 ± 14.13 years, all diagnosed with at least one vertebral compression fracture (VCF) as shown in Figure 2. The images, utilized for segmentation and classification, are T1-weighted median sagittal slices with varying fields of view (FOV) and comprises 315 vertebral bodies. The images are enhanced by applying spatial filtering (O'Neill, 1956) to adjust pixel intensities across images, the operation is formulated as *g*(*x, y*) = *T*[*f*(*x, y*)] where g is the output, *f* is the input image and T is an operation of *f* defined over some neighborhood of (*x, y*) pixels. To increase the global contrast of images histogram equalization is used to redistributes the intensity values in such a way the histogram of the grayscale image is calculated to represent the frequency of each intensity level (Abdullah-Al-Wadud et al., 2007). The cumulative distribution function (CDF) of the histogram is computed as: $CDF\left(i\right)=\overset{i}{\underset{j=0}{\sum }}h\left(j\right)$ and normalized by dividing each value by the total number of pixels *N* in the image: ${\text{CDF}}_{\text{norm}}\left(i\right)=\frac{\text{CDF}\left(i\right)}{N}$ with this normalized CDF, a mapping of the original intensity levels to new intensity levels is created: *I*_new_(*i*) = round(CDF_norm_(*i*) × (*L*−1)) where *L* is the number of possible intensity levels in digital images typically 256, as images generally store pixel brightness using 8 bits per pixel. This allocation allows for 256 distinct brightness levels, ranging from (0 to 255), ensuring smooth gradations in brightness while optimizing storage efficiency. The image is transformed by applying this mapping, resulting in an equalized image with enhanced contrast this helps to overcome image inhomogeneity, making different features more distinguishable.

### 3.2 Segmentation of vertebral bodies

Segmentation involves partitioning an image of the lumbar spine into distinct regions, where each region consists of pixels that exhibit similar characteristics. This process is essential for ensuring that each vertebral body is accurately identified and delineated. To isolate the region of interest (ROI) and eliminate the background, the binary image is processed using morphological operations. Morphological filters used are based on two main operations dilation and erosion (Maragos, 1989):


(1) $$\begin{array}{l} A⊗B=\left\{z\;|\;{\left(\hat{B}\right)}_{z}\cap A\neq \phi \right\} \end{array}$$

where ϕ is the empty set and $\hat{B}$ is the reflection of the structuring element B. Erosion is defined as:


(2) $$\begin{array}{l} A\theta B=\left\{z|{\left(B\right)}_{z}\cap A^{c}\neq \emptyset \right\} \end{array}$$

where *A*^*c*^ is a complement of *A*. Following morphological operations, object holes are filled, and regions touching the image boundaries that do not belong to the vertebra are removed, while smaller, more irregular island areas, compared to the vertebral bones, are eliminated. To ensure only the vertebral body (VB) region is retained, the centroids of all components are calculated, and only those with centroid x-coordinates within a specified threshold are kept, enabling the precise localization of VBs. Overall dataset includes both normal and fractured vertebral bodies, with severely compressed and fragmented regions categorized as background and removed during morphological operations. This removal can result in irregular boundaries, complicating shape-based analysis. To address this, the convex hull is applied, producing the smallest convex boundary around the vertebral bodies. This regularizes the shape, ensuring smoother contours while preserving the overall structure, making the data more suitable for segmentation. Preparata and Shamos (2012) The convex hull of a set of points *P* = {*p*_1_, *p*_2_, …, *p*_*n*_} in a Euclidean space is the smallest convex polygon that encloses all the points in *P*. Mathematically, the convex hull Conv(P) is defined as the set of all convex combinations of points in *P*:


(3) $$\begin{array}{l} \text{Conv}\left(P\right)=\left\{\overset{n}{\underset{i=1}{\sum }}\lambda_{i}p_{i}|\lambda_{i}\geq 0,\overset{n}{\underset{i=1}{\sum }}\lambda_{i}=1\right\} \end{array}$$

This means that any point *q* in the convex hull can be expressed as a convex combination of the points in *P*, where the coefficients λ_*i*_ are non-negative and sum up to 1:


(4) $$\begin{array}{l} q=\overset{n}{\underset{i=1}{\sum }}\lambda_{i}p_{i} \end{array}$$

In the context of vertebral body segmentation, the convex hull is used to create a convex polygon that encloses the segmented region (vertebral bodies) after morphological operations. This ensures that the region of interest, corresponding to vertebral bodies present in the lumbar spine, is accurately enclosed, excluding severely compressed and fragmented regions categorized as background.

### 3.3 Classification

The objective of an automated medical image classification system is to develop a model that can efficiently and accurately categorize images as either normal or abnormal (Deepa et al., 2011). In this study we aim to classify MRI spine images into normal or fractured categories. Vertebral irregularities are predominantly observed in the vertebral body (VB). Benign fractures typically alter the shape of the VB while maintaining surface similarity to normal VBs, whereas malignant fractures cause more significant variations in surface textural intensities but retain structural resemblance to normal VBs. This research focuses on VBs on lumbar spine labeled L1–L5 for diagnostic purposes, utilizing a machine learning framework that considers multiple VB features such as shapes, gray level values, textures and boundaries. These features, individually or collectively, are employed in various medical applications, including ROI localization, abnormality detection with specific patterns, anatomical structure segmentation, and differentiation between healthy and pathological instances.

#### 3.3.1 Features extraction (FE)

Feature extraction refers to the process of transforming data into a set of meaningful attributes (features) that are informative and non-redundant, aiding in learning and generalization. This technique is often employed to simplify large datasets by reducing the number of input variables while retaining critical information. Unlike dimensionality reduction, feature extraction focuses on selecting characteristics that enhance the performance of subsequent algorithms. The efficacy of any algorithm is closely tied to the quality of its feature detector, which influences the overall performance of the system. As highlighted by Caridakis et al. (2008) , a feature is the “interesting" portion of an image that underpins the success of subsequent computational procedures Complex data analysis faces significant challenges due to the high dimension of variables, which can lead to excessive memory and computational demands. Furthermore, a large number of variables can cause classification algorithms to overfit to the training samples and results generalize poorly to new data (Liu and Gillies, 2016). Consequently, the desirable property for a feature detector is its repeatability, i.e., the ability to detect the same feature in multiple images of the similar scene. The primary types of features considered in sign identification are spatial, temporal, and textural (Choras et al., 2007). The feature extraction stage is designed to process real images, with algorithms typically divided into three tasks: extraction, selection, and classification. For classification to be valid, there must be a logical connection between the features, as the specific features available for discrimination directly impact the classification's effectiveness. Feature extraction refers to various methods for combining variables to address these issues while sufficiently describing the data. Many ML practitioners believe that well-optimized feature extraction is crucial for effective model construction. The process of feature extraction results in a collection of characteristics, often called a feature vector, which encapsulates the essential information of an image.

#### 3.3.2 Shape features

Shape is an important visual feature of an image that needs to be described or represented by certain characteristics. Methods for recognizing shapes in images can be categorized into edge-based, region-based, or feature-based approaches. Edge-based methods identify edge points by detecting intensity discontinuities, followed by applying closing and filtering operations to form distinct shapes (Wang and Yang, 2012). Effective shape features must exhibit certain essential properties, including being identifiable, invariant to translation, rotation, and scale, as well as affine and occlusion invariance (Zhang and Lu, 2004). Additionally, they should resist noise and be statistically independent. Shape-based image retrieval involves measuring the similarity between shapes based on their features. Simple geometric features effectively capture and describe shapes with notable differences. These features serve as efficient filters to exclude irrelevant matches and are often complemented by more advanced shape descriptors to enhance precision in complex shape discrimination. Key shape parameters include the center of gravity, axis of least inertia, digital bending energy, eccentricity, circularity ratio, elliptical variance, rectangularity, convexity, solidity, Euler number and profiles (Mingqiang et al., 2008). One dimensional function for shape representation also known as shape signature derived from boundary coordinates.Shape signatures usually captures the perceptual feature of shape, the centroid distance function, area function and chord length function. In this study, we utilize the following shape features:

Center of Gravity also called the centroid. Its position should be fixed in relation to the shape. The shape is represented by its region function its centroid (*g*_*x*_, *g*_*y*_) is:


(5) $$\{\begin{array}{l} g_{x}=\frac{1}{N}\sum_{i=1}^{N}x_{i}\\ g_{y}=\frac{1}{N}\sum_{i=1}^{N}y_{i} \end{array}$$

where *N* is the number of points in the shape, (*x*_*i*_, *y*_*i*_)∈{(*x*_*i*_, *y*_*i*_)∣*f*(*x*_*i*_, *y*_*i*_) = 1}.

Axis of least inertia this is a unique line for a given shape that acts as a reference to maintain the shape's orientation.

Average bending energy (BE) is defined by


(6) $$\begin{array}{l} \text{BE}=\frac{1}{N}\overset{N-1}{\underset{s=0}{\sum }}K{\left(s\right)}^{2} \end{array}$$

where *K*(*s*) represents the curvature function, *s* is the arc length parameter, and *N* is the number of points on the contour. In order to compute the average bending energy more efficiently, Young et al. (1974) performed the Fourier transform of the boundary and used Fourier coefficients and Parseval's relation.

Eccentricity refers to the aspect ratio, which is the ratio of the length of the major axis to the length of the minor axis, typically determined using the principal axes method.

Principal axes of a given shape are the two lines that intersect orthogonally at the centroid of the shape. These axes represent directions with zero cross-correlation, meaning they are statistically independent in terms of distribution (Peura et al., 1997). This way, a contour is seen as an instance from a statistical distribution. Let us consider the covariance matrix *C* of a contour:


(7) $$\begin{array}{l} C=\frac{1}{N}\overset{N-1}{\underset{i=0}{\sum }}\left(\begin{array}{c} x_{i}-g_{x}\\ y_{i}-g_{y} \end{array}\right){\left(\begin{array}{cc} x_{i}-g_{x} & y_{i}-g_{y} \end{array}\right)}^{T}=\left(\begin{array}{cc} c_{xx} & c_{xy}\\ c_{yx} & c_{yy} \end{array}\right) \end{array}$$

where


(8) $$\begin{array}{ll} c_{xx} & =\frac{1}{N}\overset{N-1}{\underset{i=0}{\sum }}{\left(x_{i}-g_{x}\right)}^{2} \end{array}$$

(9) $$\begin{array}{ll} c_{xy} & =\frac{1}{N}\overset{N-1}{\underset{i=0}{\sum }}\left(x_{i}-g_{x}\right)\left(y_{i}-g_{y}\right) \end{array}$$

(10) $$\begin{array}{ll} c_{yx} & =\frac{1}{N}\overset{N-1}{\underset{i=0}{\sum }}\left(y_{i}-g_{y}\right)\left(x_{i}-g_{x}\right) \end{array}$$

(11) $$\begin{array}{ll} c_{yy} & =\frac{1}{N}\overset{N-1}{\underset{i=0}{\sum }}{\left(y_{i}-g_{y}\right)}^{2} \end{array}$$

*G*(*g*_*x*_, *g*_*y*_) is the centroid of the shape. Clearly, here *c*_*xy*_ = *c*_*yx*_.

The lengths of the two principal axes equal the eigenvalues λ_1_ and λ_2_ of the covariance matrix *C* of a contour, respectively. So the eigenvalues λ_1_ and λ_2_ can be calculated by


(12) $$\begin{array}{l} \begin{array}{cc} \det\left(C-\lambda_{1,2}I\right) & =\det\left(\begin{array}{cc} c_{xx}-\lambda_{1,2} & c_{xy}\\ c_{yx} & c_{yy}-\lambda_{1,2} \end{array}\right)\\ =\left(c_{xx}-\lambda_{1,2}\right)\left(c_{yy}-\lambda_{1,2}\right)-c_{xy}^{2}=0 \end{array} \end{array}$$

So


(13) $$\begin{array}{l} \begin{array}{cc} \lambda_{1} & =\frac{1}{2}\left(c_{xx}+c_{yy}+\sqrt{{\left(c_{xx}+c_{yy}\right)}^{2}-4\left(c_{xx}c_{yy}-c_{xy}^{2}\right)}\right)\\ \lambda_{2} & =\frac{1}{2}\left(c_{xx}+c_{yy}-\sqrt{{\left(c_{xx}+c_{yy}\right)}^{2}-4\left(c_{xx}c_{yy}-c_{xy}^{2}\right)}\right) \end{array} \end{array}$$

Then, eccentricity determined by the ratio of the eigenvalues:


(14) $$\begin{array}{l} E=\frac{\lambda_{2}}{\lambda_{1}} \end{array}$$

Rectangularity quantifies how well a shape approximates a rectangle, specifically how much the shape fills its minimum bounding rectangle. It is defined as:


(15) $$\begin{array}{l} \text{Rectangularity}=\frac{A_{S}}{A_{R}} \end{array}$$

where *A*_*S*_ is the area of a shape and *A*_*R*_ is the area of its minimum bounding rectangle.

Convexity measures the ratio of the perimeter of the convex hull *O*_Convexhull_ to the perimeter of the original contour *O*:


(16) $$\begin{array}{l} \text{Convexity}=\frac{O_{\text{Convexhull}}}{O} \end{array}$$

Euler number provides a measure of the topology of the shape, specifically the relationship between the number of contiguous parts *S* and the number of holes *N* in the shape. It is number is calculated as:


(17) $$\begin{array}{l} \text{Eul}=S-N \end{array}$$

Profiles are the projection of the shape to the x-axis and y-axis on a Cartesian coordinate system. The two one-dimension functions:


(18) $$\begin{array}{l} P_{rox}\left(i\right)=\overset{j_{\text{max}}}{\underset{j=j_{\text{min}}}{\sum }}f\left(i,j\right) \end{array}$$

and


(19) $$\begin{array}{l} P_{roy}\left(j\right)=\overset{i_{\text{max}}}{\underset{i=i_{\text{min}}}{\sum }}f\left(i,j\right) \end{array}$$

where *f*(*i, j*) represents the region of the shape.

#### 3.3.3 Data augmentation

**Figure 3:**
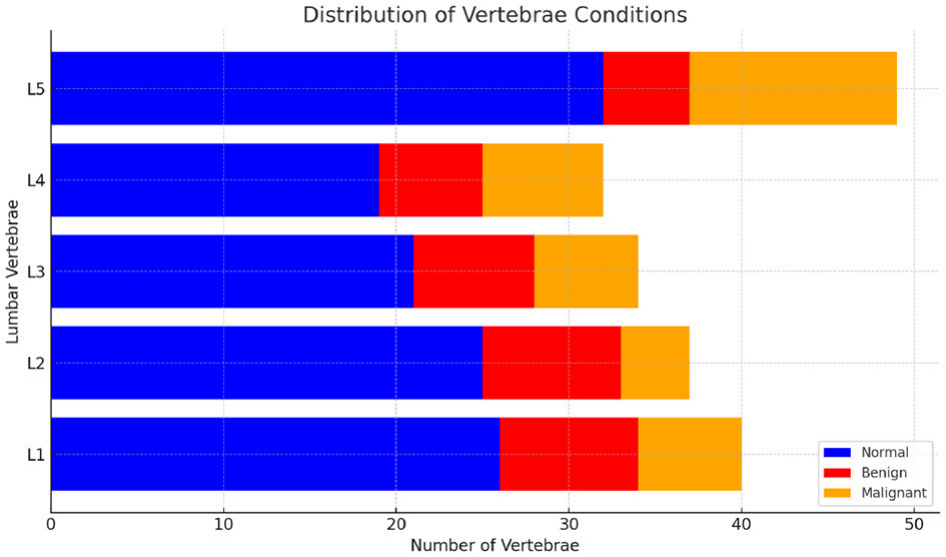
Distribution of vertebrae condition normal, benign and malignant.

For classification purposes, each segmented vertebral body (VB) is analyzed as an independent entity with its specific features extracted. In medical imaging, class imbalance is a common challenge where the number of images representing abnormal conditions (such as vertebral compression fractures, VCFs) is significantly lower than the number of images representing normal conditions. the distribution of normal and fractured (benign and malignant) vertebrae in the dataset are shown in Figure 3. This imbalance can result in machine learning models that are biased toward the majority class, leading to poor performance in detecting and classifying the minority class. To address the class imbalance in dataset, data augmentation technique Synthetic Minority Over-sampling Technique (SMOTE). Chawla et al. (2002) was employed to generate synthetic images of the minority classes i.e. fractured, thereby increasing their representation. Additionally, data augmentation techniques were applied, including rotations between -15 and 15 degrees, to further diversify the dataset. This combined approach of SMOTE and data augmentation effectively mitigated class imbalance.

## 4 Experimental evaluation and results

### 4.1 Evaluation of segmentation performance

The segmentation performance was evaluated using a leave-eleven-out cross-validation approach. In each iteration, 11 of the 63 cases were sequentially set aside for testing, while the remaining cases were used for training. This method was specifically chosen to balance the need for sufficient training data while still allowing for a robust evaluation on a significant portion of the dataset. The leave-eleven-out approach, compared to traditional k-fold cross-validation, offers a compromise between bias and variance. While k-fold cross-validation typically provides a broader view by averaging performance over multiple folds, the leave-eleven-out method was selected here to maintain a relatively large training set, which is critical given the modest dataset size. This method also ensures that each case is used multiple times across different test sets, thereby reducing variance in the performance metrics.

To minimize bias in the outcomes, the cross-validation procedure was repeated across 6 folds. The final fold comprised three fewer cases due to the dataset's size, ensuring that all cases were utilized. During each fold, the segmented outputs of the test data were compared against their corresponding ground truth segmentations. For each test case, quantitative metrics such as accuracy (Equation 20), sensitivity (Equation 21), Dice Similarity Coefficient (DSC) (Equation 22), and Jaccard Coefficient (JC) (Equation 23) were examined.

The chosen leave-eleven-out cross-validation method provides a robust evaluation strategy that carefully balances the bias-variance trade-off. This is especially pertinent for this dataset, where a smaller number of cases could lead to higher variance if a traditional k-fold approach with fewer folds was employed. All implementations were performed using MATLAB (MATLAB, 2023; version r2023a) on a 64-bit operating system with an Intel^®^ Core™ i7-10750H CPU @ 2.60 GHz.

Accuracy of segmentation *A*(*seg*) is defined as the ratio of correctly classified pixels (both true positives and true negatives) to the total number of pixels, given as:


(20) $$\begin{array}{l} A_{\text{seg}}=\frac{TP+TN}{TP+FP+TN+FN}\% \end{array}$$

Sensitivity (*S*_seg_) measures the proportion of the region of interest (ROI) that is correctly identified and is given by the equation:


(21) $$\begin{array}{l} S_{\text{seg}}=\frac{TP}{TP+FN}\% \end{array}$$

Dice Similarity Coefficient (*DSC*_seg_) measures the extent of overlap between the automatically generated segmentation and the manually annotated segmentation.


(22) $$\begin{array}{l} DSC_{\text{seg}}=\frac{2\times TP}{2\times TP+FP+FN}\% \end{array}$$

**Figure 4:**
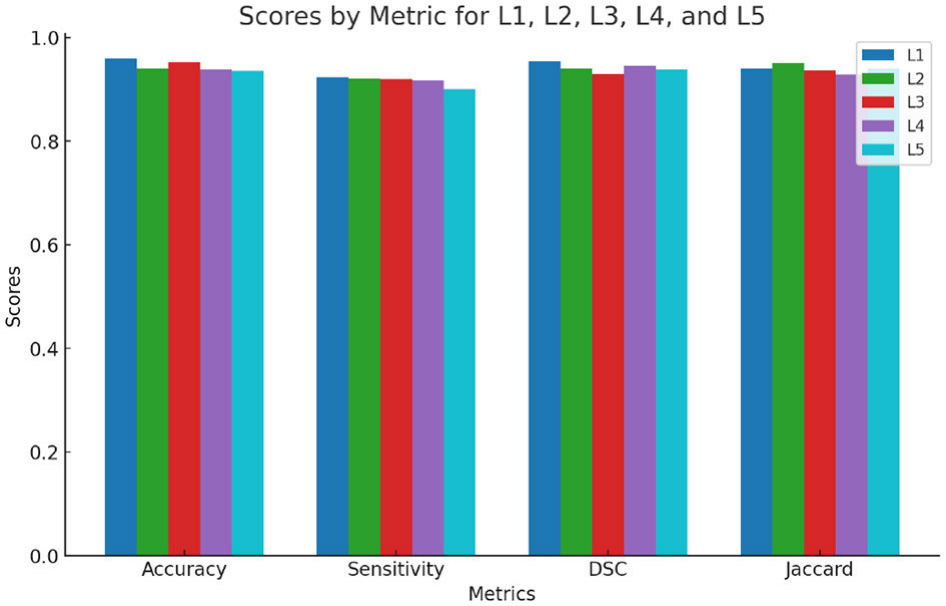
Segmentation performance evaluated at each lumbar level (L1–L5).

Jaccard Coefficient (*JC*_seg_) evaluates the similarity between the automated segmentation and the manual segmentation:


(23) $$\begin{array}{l} JC_{\text{seg}}=\frac{TP}{TP+FP+FN}\% \end{array}$$

where, TP refers to True Positive, indicating correctly identified regions of interest (ROI). TN stands for True Negative, which represents correctly identified non-ROI areas. FP denotes False Positive, where non-ROI regions were mistakenly identified as ROI, and FN stands for False Negative, indicating that ROI regions were incorrectly classified as non-ROI. Figure 4 presents the segmentation outcomes corresponding to these metrics.

### 4.2 Comparative analysis of KNN and SVM for classification tasks

This study involves a classification task aimed at identifying whether vertebral bodies (VB) are normal or fractured. There are two classes in this classification task. To measure the goodness of the proposed model computed classification accuracy, precision, recall and F-measure are computed Precision which measures the proportion of correctly predicted positive instances (true positives, TP) out of all instances predicted as positive (true positives plus false positives). It quantifies the model's ability to avoid false positives, FP (Powers, 2020).


(24) $$\begin{array}{l} \text{Precision}=\frac{\text{TP}}{\text{TP + FP}} \end{array}$$

Recall, also known as sensitivity or true positive rate, measures the proportion of correctly predicted positive instances (true positives) out of all actual positive instances (true positives plus false negatives). It quantifies the model's ability to identify all positive instances (Powers, 2020).


(25) $$\begin{array}{l} \text{Recall}=\frac{\text{TP}}{\text{TP + FN}} \end{array}$$

Accuracy measures the overall correctness of the model's predictions by calculating the ratio of correctly classified instances to the total number of instances in the dataset (Powers, 2020). It provides a general assessment of the model's performance.


(26) $$\begin{array}{l} \text{Accuracy}=\frac{\text{TP + TN}}{\text{TP + TN + FP + FN}} \end{array}$$

The F1 score (Powers, 2020) is a widely used metric that combines precision and recall to provide a balanced measure of the model's performance. It considers both the false positives and false negatives, making it suitable for imbalanced datasets.


(27) $$\begin{array}{l} \text{F1 Score}=\frac{2\times \text{Precision}\times \text{Recall}}{\text{Precision}+\text{Recall}} \end{array}$$

The average of classification accuracy using KNearest Neighbor classifier and SVM are tabulated.

#### 4.2.1 K-nearest neighbor

The k-nearest neighbor (k-NN) algorithm is a non-parametric technique utilized for both classification and regression tasks.The algorithm works by identifying the “k" closest data points in the feature space to make predictions about a new, unseen data point. For classification, the algorithm assigns a class to the new data point based on a majority vote among its k nearest neighbors. The process involves first calculating the distance between the input data point and all points in the training set, typically using the Euclidean distance, defined as


(28) $$\begin{array}{l} d\left(x,x^{\prime }\right)=\sqrt{{\left(x_{1}-x_{1}^{\prime }\right)}^{2}+\ldots +{\left(x_{n}-x_{n}^{\prime }\right)}^{2}} \end{array}$$

where *x* and *x*′ are two data points in an n-dimensional space. The input gets assigned to the class with the largest probability given as:


(29) $$\begin{array}{l} P\left(y=j|X=x\right)=\frac{1}{K}\underset{i\in A}{\sum }I\left(y^{\left(i\right)}=j\right) \end{array}$$

The performance of the k-NN algorithm is highly sensitive to the choice of *k*, as a smaller *k* may produce noisy decision boundaries, while a larger *k* could overly smooth these boundaries, potentially missing important local patterns. Thus, the k-NN algorithm leverages the proximity of data points to make predictions based on the most similar examples from the training set, making it a simple yet effective method for classification and regression (Altman, 1992). To optimize the k-Nearest Neighbors (kNN) classifier for classifying normal and fractured vertebrae, a grid search across different values of *k* given (*k* = 3, *k* = 5, *k* = 7, *k* = 9, *k* = 11) was conducted. The performance metrics considered include accuracy, precision, recall, and F-measure, with various train-test splits (20:80, 30:70, 40:60, 50:50, 60:40, 70:30, 80:20). The results for each k-value are presented in Table 1 , followed by a comparative analysis of the models in terms of accuracy and precision to highlight the best-performing one. The analysis revealed that *k* = 3 provided the best overall performance, with the highest average accuracy of 84.78%, precision of 87.88%, recall of 90.37%, and an F-Measure of 88.63%, underscoring the importance of carefully selecting the value of *k* to ensure the algorithm's robustness and reliability in accurately classifying both normal and fractured vertebrae.

**Table 1:** Performance metrics for K-NN with different k-values and train-test splits.

**Train%-Test%**	**Accuracy**	**Precision**	**Recall**	**F-Measure**
***k*** **= 3**				
30–70	74.55	77.65	86.45	81.63
40–60	77.89	80.55	88.09	84.04
50–50	80.97	85.50	87.92	86.70
60–40	85.20	87.60	90.10	88.65
70–30	93.16	96.02	93.67	94.52
80–20	**96.92**	**98.89**	95.97	**97.25**
**Average**	84.78	87.88	90.37	88.63
***k*** **= 5**				
30–70	73.41	74.77	90.44	81.74
40–60	75.79	76.40	91.79	83.25
50–50	78.71	79.56	93.04	85.54
60–40	76.00	78.11	90.09	83.47
70–30	80.53	82.16	92.41	86.57
80–20	81.54	86.27	89.74	87.60
**Average**	77.66	79.55	91.25	84.69
***k*** **= 7**				
30–70	73.18	71.91	**96.26**	82.03
40–60	76.32	76.20	94.68	84.04
50–50	78.39	79.70	93.26	85.78
60–40	80.40	83.59	90.39	86.67
70–30	82.11	82.89	96.03	88.81
80–20	86.15	91.71	91.86	91.49
**Average**	79.42	81.00	93.75	86.47
***k*** **= 9**				
30–70	75.23	72.95	**98.28**	83.57
40–60	75.00	73.87	96.06	83.41
50–50	78.71	80.32	92.85	85.80
60–40	82.40	83.31	94.45	88.39
70–30	84.21	88.56	91.98	89.92
80–20	**91.54**	91.28	99.09	**94.92**
**Average**	81.18	81.71	95.45	87.67
***k*** **= 11**				
30–70	74.77	73.49	96.54	83.33
40–60	75.26	73.52	**97.36**	83.60
50–50	77.74	76.95	96.21	85.38
60–40	80.40	81.60	93.94	87.14
70–30	81.58	87.06	97.80	92.18
80–20	87.69	89.95	95.17	92.11
**Average**	79.57	79.38	96.17	86.65

The best performance for each metric is highlighted in bold.

#### 4.2.2 Support vector machines (SVM)

Support Vector Machines (SVM) are a robust technique for constructing classifiers. The primary goal is to establish a decision boundary between two classes that facilitates the prediction of labels from one or more feature vectors (Noble, 2006). This decision boundary, termed the hyperplane, is optimally placed to be as far as possible from the closest data points of each class, which are termed support vectors. For a labeled dataset:


(30) $$\begin{array}{l} {\left\{\left(x_{i},y_{i}\right)\right\}}_{i=1}^{n} \end{array}$$

where *x*_*i*_ is the feature vector and *y*_*i*_ represents the class label for a training sample *i*.

The hyperplane can be defined as:


(31) $$\begin{array}{l} w\cdot x+b=0 \end{array}$$

where *w* is the weight vector, *x* is the input feature vector, and *b* is the bias term.

The vectors *w* and *b* must satisfy the following conditions for all training samples:


(32) $$\begin{array}{l} y_{i}\left(w\cdot x_{i}+b\right)\geq 1 \end{array}$$

The goal of training an SVM model is to find *w* and *b* such that the hyperplane divides the data while maximizing the margin


(33) $$\begin{array}{l} \frac{1}{||w|{|}^{2}} \end{array}$$

Vectors *x*_*i*_ for which $|y_{i}|\left(wx_{i}^{T}+b\right)=1$ will be termed support vectors.

The optimization of Support Vector Machine (SVM) for classification of normal and fractured vertebrae, model's performance was maximized, through tuning the regularization parameter C, which controls the trade-off between margin maximization and classification error minimization. A thorough grid search with cross-validation was employed to identify the optimal C value, ensuring that the model achieved strong generalization without overfitting. Focusing on the linear kernel allowed for the streamlining of the hyperparameter tuning process while reducing computational complexity.

The performance metrics of a linear Support Vector Machine (SVM) applied to the classification task are detailed in Table 2, showcasing results across various train-test splits. The SVM consistently performs well, with the highest accuracy 98.00%, precision98.16%, recall 98.86%, and F-measure 98.46% observed in the 60–40 split, indicating exceptional generalization and detection capabilities. The 50–50 split also shows remark- able performance, with metrics nearly matching those of the 60–40 split, suggesting that a balanced train-test ratio may optimize the SVM's performance.

**Table 2:** Performance metrics for linear SVM.

**Train%-Test%**	**Accuracy**	**Precision**	**Recall**	**F-Measure**
30–70	95.72	96.36	97.10	96.54
40–60	96.19	96.24	97.60	96.89
50–50	97.42	98.10	98.04	98.02
60–40	**98.00**	98.16	**98.86**	**98.46**
70–30	96.58	97.27	95.53	96.69
80–20	98.15	**98.89**	95.53	97.11
**Average**	**97.01**	**97.50**	**97.11**	**97.28**

The best performance for each metric is highlighted in bold.

#### 4.2.3 Performance comparison between KNN and SVM

The performance of KNN and SVM classifiers was statistically analyzed using paired t-tests and Wilcoxon signed-rank (Blair and Higgins, 1985) tests to determine the significance of differences in their accuracy scores across various configurations. Accuracy values were obtained for KNN with *k* = 3, 5, 7, 9, and 11. compared against SVM accuracies.

These results show that SVM significantly outperforms KNN for all tested values of k, as indicated by the paired *t*-test *p*-values being < 0.05. The SVM classifier exhibits higher and more consistent accuracy scores compared to KNN, with statistically significant differences observed for all KNN configurations. According to the Wilcoxon signed-rank test, the accuracy differences between SVM and KNN configurations with *k* = 3, 5, 7, 9, 11, and 13 are statistically significant *p* < 0.05 as shown in Table 3. However, for KNN with *k* = 3, while the paired *t*-test shows statistical significance, the Wilcoxon signed-rank test presents a *p*-value of 0.03125, which still indicates a significant difference. Despite the statistical significance, KNN with *k* = 3 shows performance that is relatively close to that of SVM. This suggests that KNN with *k* = 3 could be a viable alternative in contexts where SVM might be too complex or resource-intensive, especially considering factors such as computational efficiency or ease of implementation.

**Table 3:** Statistical test results comparing KNN (with various *k*) and SVM.

**Comparison**	**Paired t-test(*t-*statistic, *p*-value)**	**Wilcoxon signed-rank test (*w*-statistic, *p*-value)**
KNN (*k* = 3) vs SVM	-3.67, **0.0145**	0.0, **0.03125**
KNN (*k* = 5) vs SVM	-17.74, **1.05e-05**	0.0, **0.03125**
KNN (*k* = 7) vs SVM	-11.29, **9.52e-05**	0.0, **0.03125**
KNN (*k* = 9) vs SVM	-6.95, **0.00095**	0.0, **0.03125**
KNN (*k* = 11) vs SVM	-10.41, **0.00014**	0.0, **0.03125**

The *p*-values indicating significant differences *p* < 0.05 are highlighted in bold.

**Figure 5:**
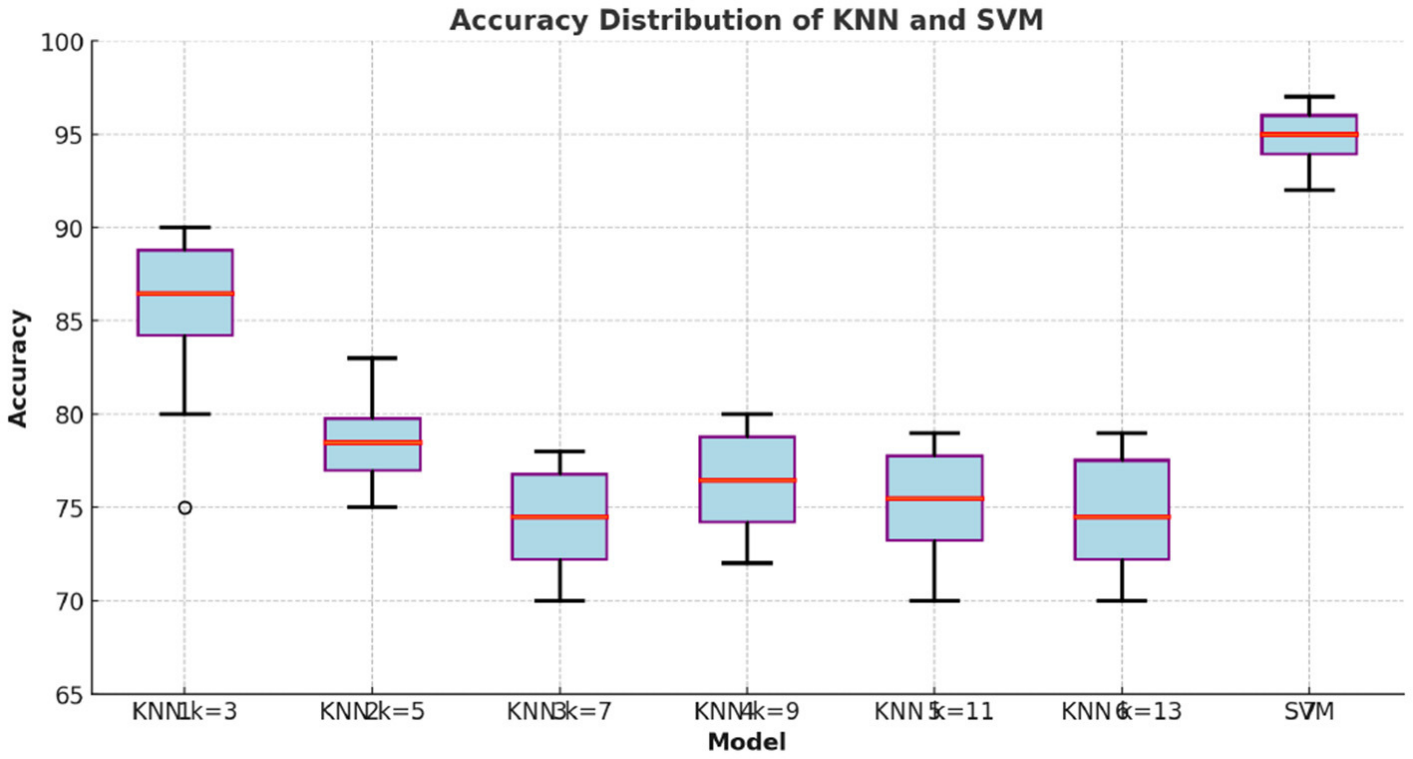
Comparison of the accuracy distribution between KNN and SVM.

Figure 5 illustrates the distribution of accuracy values for different KNN *k* = 3, 5, 7, 9, and 11 configurations and SVM. The SVM model demonstrates higher median accuracy and lower variability compared to all KNN configurations, indicating more consistent performance. In contrast, the KNN models show greater variability in accuracy, with generally lower median values. As k increases, the performance of KNN improves, but it still does not reach the levels achieved by SVM. This visual representation supports the statistical test results, highlighting the superior and stable performance of the SVM classifier across different experimental runs.

## 5 Discussion

The proposed framework for segmentation and classification effectively distinguishes skeletal structures from soft tissues in MR images, demonstrating robust accuracy and computational efficiency. It involves three critical steps: data preprocessing, segmentation of lumbar vertebral bodies (VBs), and feature extraction for classification. The preprocessing step, employing spatial filtering and histogram equalization, effectively enhances gray level uniformity and image contrast, addressing issues of image inhomogeneity, significantly facilitates accurate detection and localization of vertebral bodies.

The segmentation approach leverages morphological operations combined with the convex hull technique, effectively isolating regions of interest while removing background noise. This ensures precise localization of vertebral bodies, crucial for reliable feature extraction and classification. Unlike manual segmentation methods, such as described by Azevedo-Marques et al. (2015), which can introduce operator subjectivity and variability, the automated approach utilized here offers consistency, efficiency, and reduced reliance on human intervention. In terms of classification, the utilization of shape features like aspect ratio, area function, centroid distance function effectively highlight differences in contour or height distribution, which are key in distinguishing normal from fractured vertebral bodies. Compared to traditional machine learning classifiers such as the k-nearest neighbor (KNN) and support vector machines (SVM), as demonstrated in earlier studies (Frighetto-Pereira et al., 2016), our method achieves superior classification performance. Support vector machines have also shown promising results in previous literature, effectively handling non-linear classification tasks through kernel functions (Arpitha and Rangarajan, 2021). Neural network-based methods, including deep learning techniques, have become prevalent due to their powerful feature extraction capabilities. Germann et al. (2023) demonstrated excellent classification performance using neural network methodologies; however, these approaches necessitate large annotated datasets and substantial computational power, limiting their applicability in resource-constrained settings. Conversely, our method does not rely on extensive datasets or high computational demands, offering broader accessibility. Although the current study is limited to lumbar spine imaging in the sagittal plane, future work could explore the applicability of the method across the thoracic spine and different imaging modalities. Overall, the proposed methodology demonstrates notable advantages over traditional KNN, SVM, and neural network approaches by offering robust performance, reduced computational demands as shown in Table 4.

**Table 4:** Classification accuracy of different methods.

**Literature**	**Classification accuracy**
Frighetto-Pereira et al. (2016)	96%
Frighetto-Pereira et al. (2016)	97%
Arpitha and Rangarajan (2021)	94.17%
Arpitha and Rangarajan (2021)	96.07%
Yeh et al. (2022)	92.0%
Germann et al. (2023)	96.20%
Present method	97.01%

## 6 Conclusions and future work

This study applies machine learning techniques to preprocess, segment and classify lumbar vertebral fractures (VCFs) in medical images, enhancing classification metrics and diagnostic confidence. While focused on lumbar VBs, the methods can extend to thoracic, cervical and sacral VB. Future work will improve segmentation performance with semantic and instance-based techniques, an automated feature selector, and explore deep learning architectures, transfer learning, and 3D convolutional networks. Extending these concepts to 3D image analysis aims to broaden applicability and improve diagnostic and treatment outcomes for spinal injuries.

## Data Availability

The data used in this study were obtained from Frighetto-Pereira et al. (2016) and are available upon reasonable request.
